# Median Arcuate Ligament Syndrome Revealed by Splenic Infarcts

**DOI:** 10.5334/jbsr.2781

**Published:** 2022-04-29

**Authors:** Olaia Chalh, Nabil Moatassim Billah, Ittimad Nassar

**Affiliations:** 1 Ibn Sina Teaching hospital, MA

**Keywords:** splenic infarcts, median arcuate ligament syndrome, celiac artery compression

## Abstract

**Teaching Points:** Splenic infarction is an extremely rare presentation of median arcuate ligament syndrome that requires a good analysis of celiac axis on computed tomography angiography in other to make a proper diagnosis and management.

## Case History

A 50-year-old man with a history of alcohol and tobacco abuse presented with chronic and recurrent epigastric pain, weight loss, and weakness. The patient was known to receive antituberculous therapy for active pulmonary tuberculosis.

**Figure 1:**
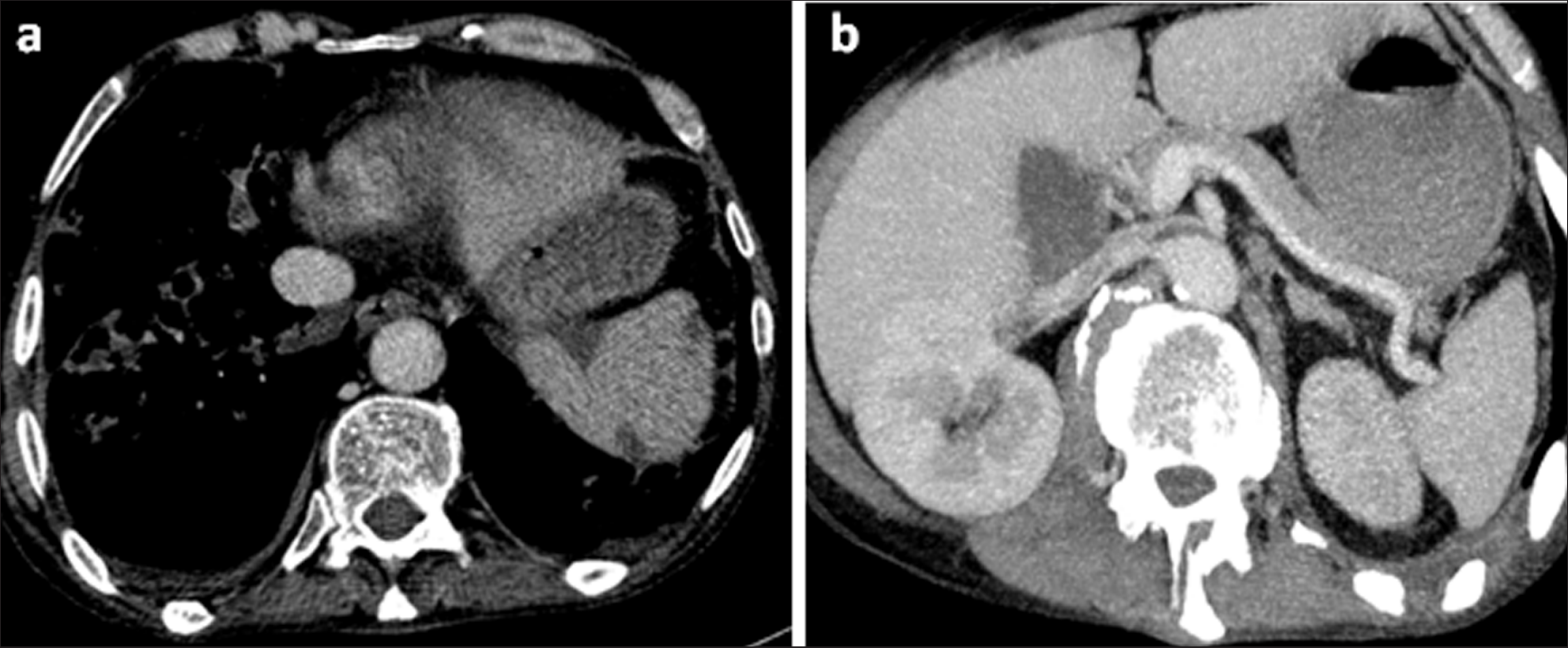


**Figure 2:**
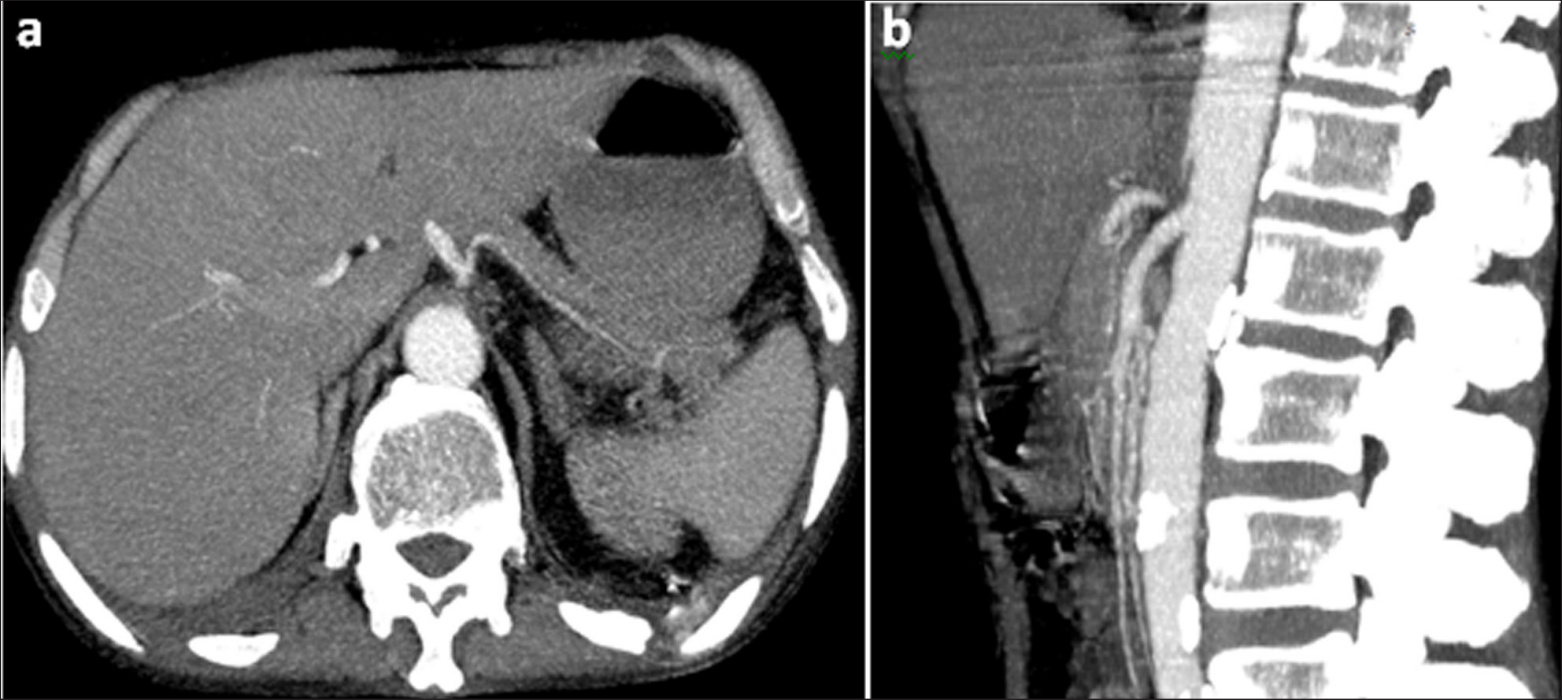


**Figure 3:**
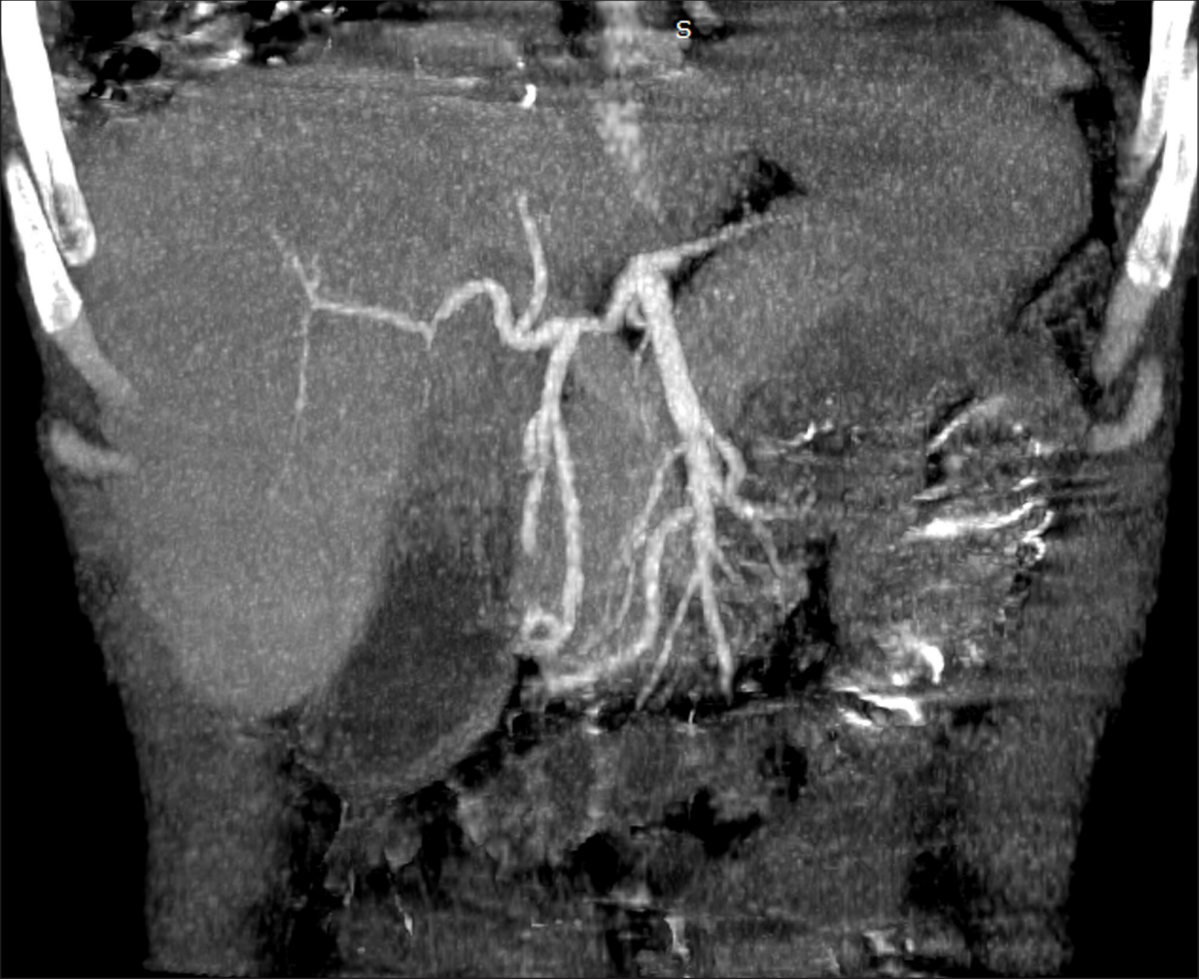


A contrast-enhanced abdominal computed tomography was performed. Axial images of portal phase demonstrated multiple wedge-shaped non-enhancing areas at the periphery of the spleen suggestive of splenic infarcts (***Figure 1a***). Splenic vein was permeable (***Figure 1b***). Axial and sagittal images of arterial phase revealed a small splenic artery related to severe stenosis and angulation of celiac axis caused by a thick soft tissue band (5 mm) anterior to the abdominal aorta creating a hooked appearance (***Figure 2a, b***). Coronal maximum intensity projection CT showed a prominent gastroduodenal artery with large collateral vessels around the pancreatic head (***Figure 3***). Median arcuate ligament syndrome causing splenic infarcts was retained and the patient was referred to vascular surgery service.

## Comments

Median arcuate ligament syndrome (MALS) is a clinical and radiological condition in which the celiac artery is compressed and inferiorly displaced by the median arcuate ligament.

Severe compression may cause mesenteric ischemia, which leads to visceral infraction. Unlike the liver, the spleen is mainly dependent on the celiac artery for its blood supply, thus, it is at greater risk for infarction during celiac occlusion. MALS occurs in approximately 2% and typically affects young patients (20–40 years of age). It is more common in thin women [1]. Clinical symptoms include postprandial epigastric pain, anorexia, and weight loss. Physical examination may reveal an abdominal bruit in the midepigastric region [1].

MALS is a diagnosis of exclusion. Conventional angiography was traditionally used for diagnosis. Nowadays, computerized tomography angiography (CTA) is the modality of choice in significant cases. True compression can be evaluated in the end-inspiratory phase. Typical findings include narrowing in the proximal celiac artery with inferior displacement resulting in characteristic hooked configuration. The sagittal plane is optimal for visualizing the proximal portion of the celiac axis. CT may also detect poststenotic dilation, large collateral vessels within the pancreatic arcade, and dilation of gastroduodenal artery. Doppler ultrasound may evaluate the severity of artery stenosis in both phases of respiration. A systolic velocity in the celiac artery more than 250 cm/s on average is highly suggestive of MALS [1].

Significant cases of MALS require surgical release of the celiac artery from compression with simultaneous removal of the nerves that are being compressed as well and endovascular stenting of the celiac artery [1].
